# Agitation Down-Regulates Immunoglobulin Binding Protein EibG Expression in Shiga Toxin-Producing *Escherichia coli* (STEC)

**DOI:** 10.1371/journal.pone.0119583

**Published:** 2015-03-06

**Authors:** Thorsten Kuczius, Wenlan Zhang, Viktor Merkel, Alexander Mellmann, Phillip I. Tarr, Helge Karch

**Affiliations:** 1 Institute for Hygiene, Westfälische Wilhelms-University and University Hospital Münster, Robert Koch-Strasse 41, 48149, Münster, Germany; 2 Department of Pediatrics, Washington University, School of Medicine, Campus Box 8208, 660 S. Euclid, St. Louis, Missouri, 63105, United States of America; The Scripps Research Institute and Sorrento Therapeutics, Inc., UNITED STATES

## Abstract

Shiga toxin (Stx)-producing *Escherichia coli* (STEC) carrying *eibG* synthesize *Escherichia coli* immunoglobulin binding protein (EibG). EibG nonspecifically binds to immunoglobulins and tends to aggregate in multimers but is poorly expressed in wild-type strains. To study synthesis of the proteins and their regulation in the pathogens, we identified natural growth conditions that increased EibG synthesis. EibG proteins as well as corresponding mRNA were highly expressed under static growth conditions while shearing stress created by agitation during growth repressed protein synthesis. Further regulation effects were driven by reduced oxygen tension, and pH up-regulated EibG expression, but to a lesser extent than growth conditions while decreased temperature down-regulated EibG. Bacteria with increased EibG expression during static growth conditions showed a distinct phenotype with chain formation and biofilm generation, which disappeared with motion. High and low EibG expression was reversible indicating a process with up- and down-regulation of the protein expression. Our findings indicate that shear stress represses EibG expression and might reduce bacterial attachments to cells and surfaces.

## Introduction

Bacterial surface proteins that interact with immunoglobulins from different mammalian species in a non-immune manner have been described for many species [1]. For example, proteins A and G synthesized by staphylococci and streptococci have well characterized immunoaffinity phenotypes [2, 3], and immunoglobulin binding proteins are found in pathogens such as *Aeromonas* [4, 5], *Pseudomonas* [6] and *Yersinia* species [7]. The conservation of these immunoglobulin binding proteins among many pathogenic bacteria suggests that these molecules might be involved in bacterial survival and/or in virulence as in streptococci [8, 9]. Immunoglobulin binding proteins (Eib proteins) are also found in *Escherichia coli*. Specifically, ECOR-9 and ECOR-2 groups express EibA, C, and D, and EibF, respectively [10, 11]. A variant, EibH, is found in *E*. *coli* strains from wild cervids [12] and EibG is present in Shiga toxin (Stx)-producing *Escherichia coli* (STEC) [13], which are a group of zoonotic pathogens [14]. The major factors of STEC that lead to pathogenesis are phage-encoded toxins Stx 1 and Stx 2 [15] that are directly implicated in both hemorrhagic and systemic STEC infections in humans, which consist of diarrhea, bloody diarrhea, and the hemolytic uremic syndrome (HUS). EibG is considered to be an additional virulence factor in STEC [13], which may also harbour further virulence factors, such as intimin, enterohemolysin, cytolethal distending toxin, a catalyse-peroxidase, and an extracellular serine protease (EspP) [16–21]. EibG seems to be involved in bacterial adherence to host epithelial cells and forms chain-like adherence patterns (CLAP), where chain length (ranging from a few cells to long chains) depends on the Eib subtype [13]. The biological function of EibG, however, is not understood.

*eibG* encodes a 508 amino acid protein that is highly similar to Eibs in other *E*. *coli*. Eibs aggregate and generate highly stable multimers that bind IgA and IgG and/or the Fc fragment of human IgG in a non-immune manner; however, interaction of Eib with the Fc fragment is not established [22–24].

Recombinant Eib proteins overexpressed in plasmids have provided most of our knowledge about these proteins [11], but the conditions and the regulation are not consistent with natural physiology.

Here, we report that EibG is naturally high expressed when host bacteria are grown under static conditions. Reduced oxygen conditions, high temperature and alkaline pH provoked an up-regulation of EibG as well, however to a lower scale than static growth. Vice versa, EibG is much less expressed when grown with shaking, low temperature and pH and with high oxygen and anaerobic growth. EibG high and low expression by strains is reversible, whereby up- and down-regulation is mainly affected by static growth or cell motion, respectively. This regulatory process might influence bacterial community phenotype as chain formation and interactions with eukaryotic cells.

## Materials and Methods

### Strains and growth experiments

EibG positive STEC [13] used in this study are listed in Table 1. Strains were obtained from the diverse strain collection at the Institute for Hygiene, Westfälische Wilhelms-University Münster and University Hospital of Münster, and at the Robert Koch Institute, Wernigerode, Germany.

**Table 1:** Oligonucleotide primers used in PCR reaction.

Target gene	Forward primers, Reverse primers	Primer sequences	Reference
*eib*G	1114orf1Fp	5′-ATC GGC TTT CAT CGC ATC AGG AC-3′	[30]
	1114orf1Rp	5′-CCA CAA GGC GGG TAT TCG TAT C-3′	
*eib*G	eibG-F1	5′-GGG TGC TTG GTG GTC TGA GTG-3′	This study
	eibG-R1	5′-CCA CAA GGC GGG TAT TCG TAT C-3′	
*eib*A	eibA-F	5′-TGA TTG GGG TGG CTT AGG TG-3′	This study
	eibA-R	5′-GTT CTT TTA TTT CAT TCG GT-3′	
*eib*C	eibC-F	5′-GGC GGT AAT GGT AAC TTC AG-3′	This study
	eibC-R	5′-ACC TTC CCA TTC TCC CCA TC-3′	
*eib*D	eibD-F	5′-AAA TAC TCC ACC TCA CAA GC-3′	This study
	eibD-R	5′-GTT ACC TGC TGC CGT GCC AT-3′	
*eib*E	eibE-F	5′-AGA AAA AGC GTC AAC ATC AG-3′	This study
	eibE-R	5′-AGA ACC ATC AAC ATA AGC AG-3′	
*eib*F	eibF-F	5′-CCA GAG AAC TCA AAA CCG CC-3′	This study
	eibF-R	5′-CCC TCT CTT TTT TAT CAG CC-3′	
*gap*A	GapA_forward	5′-GTT GTC GCT GAA GCA ACT GG-3′	[26]
	GapA_reverse	5′-AGC GTT GGA AAC GAT GTC CT-3′	

Isolates were grown in Luria broth (LB) medium or agar at 37°C. LB was used with a pH of 7.0, whereas other pH values as 5.0 for acid (supplemented with HCl) and 8.4 (with NaOH) for alkaline conditions used are indicated. For analysis, a single colony of an EibG-positive strain was pre-cultured in 13-ml-tubes with 5 ml LB with constant agitation at indicated frequencies or without shaking. Overnight cultures were inoculated at a 1:100 dilution in a volume of 30 ml LB in 100 ml or 250 ml Erlenmeyer flasks for shaking growth and for static incubation prior growth at 23°C and 37°C as indicated for 16 to 20 h. Flasks were inoculated in anaerobic jars with Anaerocult C and A (Merck, Germany) for oxygen reduction and anaerobic growth, respectively. Cells were finally pelleted by centrifugation at 3,000 rpm for 10 min at 4°C, and suspended in sterile distilled water and stored at -20°C until analysis. Protein concentrations were determined using a modified Lowry method [25].

### Molecular characterization of the strains by PCR

Strains were screened for additional *eib* genes using primers as indicated (Table 1). PCR was performed in an iCycler (Bio-Rad, Germany) in a total volume of 25 μl containing 2.5 μl of bacterial DNA purified with InstaGene Matrix (Bio-rad, Germany) as a template. The PCR conditions were: initial denaturation (94°C, 5 min), followed by 30 cycles of denaturation (94°C, 30 s), annealing (50°C for *eib*A, 52°C for *eib*C, 53°C for *eib*D and *eib*E, and 57°C for *eib*F, 60 s each), extension (72°C, 60 s) and final extension (7200B0C, 5 min). Gene products were visualized in ethidium bromide stained agarose gels.

### Quantitative reverse transcription (RT)-PCR for *eib*G mRNA expression

After growth of isolates under different conditions, total RNA of strains was isolated with the RNeasy Mini Kit (Qiagen, Germany) as recommended by the manufacturer, and treated with DNaseI (Roche, Germany). The concentration of RNA was determined by measuring optical density at 260 nm. The OD_260_ / OD_280_ nm ratios of all RNA samples were between 1.7 and 2.0, indicating a high degree of RNA purity.

A one-step quantitative RT-PCR, performed with a CFX96 (Bio-Rad, Germany) and 2x SensiMix SYBR One-Step Kit (Peqlab, Germany), measured the relative abundance of *eib*G mRNA. The PCR reactions were performed in a 96-well plate sealed with film. Reaction volumes were 20 μl and contained 1 μl of total RNA (100 ng), 10 μl of 2x SensiMix SYBR One-Step, 0.4 μl of RNase Inhibitor and 200 nM of each primer. Primers eibG-F1, eibG-R1, GapA_forward and GapA_reverse [26] were used to amplify *eib*G and *gap*A, respectively (Table 1). *gap*A encodes D-glyceraldehyde-3-phosphate dehydrogenase A and was used as a constitutively expressed reference gene for mRNA normalization [27]. The one-step RT-PCR included a reverse transcription step (42°C, 10 min), and polymerase activation and preliminary denaturation (95°C, 10 min), followed by 35 cycles of denaturation (95°C, 10s), annealing (55°C, 10s), and extension (72°C, 30s). A melting curve to confirm the specificity of the amplification products was constructed with continuous fluorescence reading from 55°C to 95°C. Data were analyzed using the Bio-Rad CFX96 standard edition optical system software V2.1, and *eib*G mRNA concentrations were normalized to *gap*A mRNA concentrations. Quantitative RT-PCRs were performed three times with two independent RNA preparations.

### Immunoblot analysis and signal quantification

Protein suspensions were added to SDS-loading buffer and heated (99°C, 5–10 min) prior to separation using sodium dodecyl sulphate (10%) polyacrylamide gel electrophoresis (SDS-PAGE) under reducing conditions in a mini slab gel apparatus (MiniProtean3 cell system; Bio-Rad, Germany). Proteins were electroblotted to polyvinylidene difluoride (PVDF) membranes (Immobilon-P; Roth, Germany) using a semidry blotting system (Roth, Germany). Membranes were blocked in Tris buffered saline (TBS) containing 0.1% Tween 20 (TBST) and 1% (w ⁄ v) nonfat dry milk powder for 60 min. To detect EibG, membranes were incubated for 16 h with horseradish peroxidase (HRP)-conjugated human IgG Fc fragment at a concentration of 100 ng/ml (Jackson ImmunoResearch Laboratories, West Grove, PA) [13]. Protein loading and regulation of EibG expression was controlled using glyceraldehyde-3-phosphate dehydrogenase (GAPDH) protein as standard marker (Sigma-Aldrich, Germany) and the polyclonal goat anti-GAPDH antibody received as a purified IgG (antibodies-online, Germany).

After intensive washing with TBST, signals were visualized with chemiluminescence enhancement (SuperSignal West Pico, Thermo Scientific, Germany) according to the manufacturer’s instructions on a chemiluminescence imager (Chemi Doc XRS, Bio-Rad, Germany) using a charge-coupled device camera. The molecular masses of the proteins were determined using the Precision Plus Protein Western C standard (Bio-Rad, Germany) according to the manufacturer’s instructions, and the signals of the standard proteins were visualized using the HRP-labelled StrepTactin antibody and chemiluminescence enhancement kit. Banding signals were quantified by densitometry and the linear range of protein signals was determined by serial dilutions [28]. Protein profiles were determined from at least three independent gels, and calculation of the signal intensities was obtained by computerized integration of the signals using the QUANTITY ONE and IMAGE LAB software (Bio-Rad, Germany). To compare data from different gels, the highest EibG signal intensities of the samples on a gel were defined as 1.0 and signal intensities of the dilutions were calculated proportionately as means. Variations in repeat SDS-PAGE runs were expressed as standard deviations of the mean (±SD). Reproducibility of high and low EibG expression was followed by several independent bacterial inoculations over multiple times. Bacteria demonstrated reproducible strain specific patterns with similar EibG protein signal intensities.

### Phenotypic appearance and microscopy

The phenotypic appearance of the cultures was inspected in Erlenmeyer flasks after growth with agitation and under static conditions at 37°C overnight (16–20h). Analysing the chain shape, phenotypical positive EibG strains were cultivated at conditions as indicated at 37°C overnight. Approximately 2x10^8^ cells were added to glass slides (Langenbrinck, Germany). After removing non-adherent bacteria, slides were washed with phosphate buffered saline. Bacteria were fixed to the surface by heating, stained with crystal violet solution (Merck, Germany), and examined by light microscopy (Axio Imager.A1; Zeiss, Germany) by an observer blinded to the identity of the isolate used. For biofilm determination, planktonic cells were removed from the Erlenmeyer tubes and the biofilm was stained with 0.1% crystal violet for 10 min [29]. After rinsing with water, attached crystal violet was documented by photography.

## Results

### EibG is highly expressed under static growth conditions

The 12 STEC used (Table 2) were confirmed to contain *eib*G; three also contained *eib*C.

**Fig 1:**
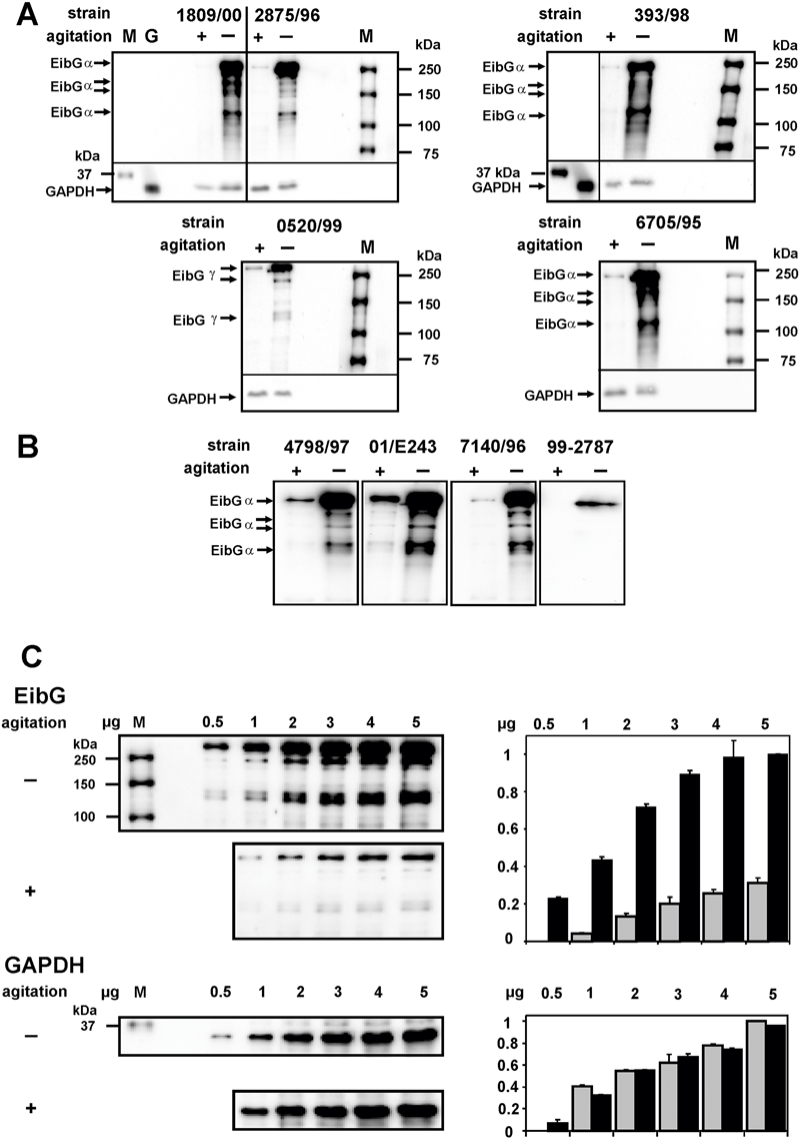
EibG expression under static growth conditions and with agitation. Cells of several STEC strains carrying the *eib*G gene were inoculated in LB medium with (+) and without (-) shaking at 37°C for 16h. Proteins were separated by SDS-PAGE and immunoblotted. (A, B) To compare expression levels, identical protein quantities of 7.5 μg were loaded in each lane. EibG was detected with human IgG Fc conjugated with HRP on immunoblots and visualized by chemiluminescence. Glyceraldehyde-3-phosphate dehydrogenase (GAPDH) was used as marker protein and as control for loading. Marker sizes (M) and EibG proteins are indicated. (C) Proteins of strain 0520/99 were diluted serially as indicated and immunoblotted to demonstrate specifity and sensitivity to the detection platform. To standardize between immunoblots, the highest intensities were defined as 1.0 and the ratios of the diluted signals of three independent gel runs were calculated as means (± standard deviations of the means). Intensities of static grown bacteria and agitated cultures are shown by black and grey bars, respectively.

EibG was highly expressed following static growth conditions with all strains analyzed in this study (Fig. 1), and poorly expressed or undetectable when bacteria were grown with agitation, as reflected by the presence or absence of a dominant band at a molecular mass > 250 kDa. The high molecular mass signals appear to reflect multimeric complexed EibG in *E*. *coli* [13]. Aggregated isoforms of monomers were also detected at lower molecular masses, though with weaker intensities, and a single distinct band was present at approximately 120 kDa (Fig. 1A, B). In comparison, GAPDH intensities were nearly invariant (Fig. 1A).

**Table 2:** STEC strains used in this study.

strain no.	serotype^a^	*eibG* subtype	*eib* genes					
			*eibA*	*eibC*	*eibD*	*eibE*	*eibF*	*eibG*
4798/97–1	O146:H21	α	-	-	-	-	-	+
3671/97	O91:H^-^ [H14]	α	-	+	-	-	-	+
393/98	O91:H^-^ [H14]	α	-	-	-	-	-	+
1809/00	O91:H14 [H14]	α	-	-	-	-	-	+
01/E243	O91:H^-^ [H14]	α	-	-	-	-	-	+
6561/95	Ont:H- [H14]	α	-	+	-	-	-	+
172/98	OR:H^-^	α	-	+	-	-	-	+
6705/95	OR:H14 [H14]	α	-	-	-	-	-	+
2875/96	O91:H14 [H14]	α	-	-	-	-	-	+
7140/96	O91:H- [H14]	α	-	-	-	-	-	+
99–02787	OR:H10	α	-	-	-	-	-	+
0520/99	Ont:H30	γ	-	-	-	-	-	+
ECOR-2	Ont:H32	-	-	-	-	-	+	-
ECOR-9	Ont:H^-^	-	+	+	+	+	-	-
659/97	O157:H-	-	-	-	-	-	-	-

^a^ [H14], strains possess *fliC* H14

Bacteria grown under static conditions produced highly intense EibG signals. Under shear stress conditions, strains expressed considerably less or no EibG. To attempt to quantify the differences in expression proteins were serially diluted and electrophoretically separated and immunoblotted to detect the EibG band (Fig. 1C). As an example, strain 0520/99 produced clear EibG signals at protein concentrations > 0.5 μg per lane after static growth. Saturation of the signal occurred between 2 and 3 μg protein. The EibG expression of agitated strains was clearly diminished, as manifest by a less intense protein signal. EibG detection of the γ-type strain 0520/99 limited at 1 μg protein per lane (Fig. 1C).

### Different EibG types

The α-, β- and γ-subtype isoforms of EibG vary in structure by several amino acids [13]. Specific protein immunoreactivity was verified with the human IgG Fc fragment while immunoglobulins of other species (e.g., goat) did not bind (not shown). Aggregated proteins were detected with a dominant band at a molecular mass > 250 kDa and a faint band at approximately 120 kDa, as seen with wild-type strains. The subcloned γ-type EibG band from 0520/99 has a higher molecular mass than the α-type from strain 1809/00. This result suggests isoform specific multimerization.

### Down-regulation of EibG by agitation

**Fig 2:**
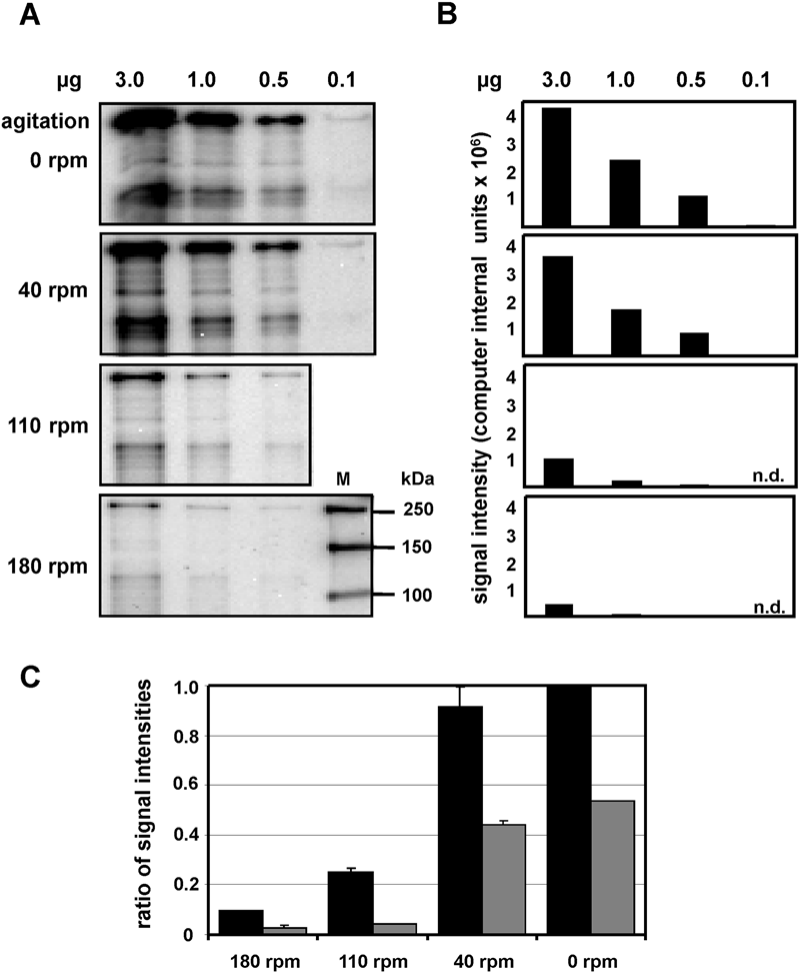
EibG expression is down-regulated by shear stress. STEC strain 2875/96 was grown in LB medium at varying revolutions per minute (rpm), as indicated. After overnight inoculation at 37°C, bacteria were harvested by centrifugation. For comparison, identical protein quantities, ranging from 3.0 to 0.1 μg per lane were separated by SDS-PAGE and immunoblotted. EibG signals were visualized using mab human Fc fragment and chemiluminescence (A). Marker sizes are indicated (M). EibG signals were quantified densitometrically and intensities are provided (B; n.d. = not determined). To analyse differences in signal intensities among protein amounts loaded on the gel and among gel runs, EibG signals of three independent immunoblots were quantified (C). The highest signal intensity was defined as 1.0 and the ratios of lower signals were estimated and reported as means (± standard deviations). The ratios of EibG signals are provided for 3 and 1 μg protein (black bars and grey bars, respectively).

Incubation of the strains in identical volume flasks (100 ml), the EibG expression varied inversely with the agitation frequency (Fig. 2). At 180 rpm, we identified modest synthesis of EibG from strain 2875/96 (Fig. 2A) with faint but visible bands diminishing in signal intensities with decreasing protein amounts (Fig. 2B), and more prominent signals with reduced agitation rates (110 to 40 rpm). The strongest signals were observed for bacteria grown under static conditions, and immunoblots showed a slight decrease in EibG signals at low rates of agitation (40 rpm) (Fig. 2C). High frequency shaking (110–180 rpm) elicited the weakest signal intensities.

### Up- and down-regulation of EibG

**Fig 3:**
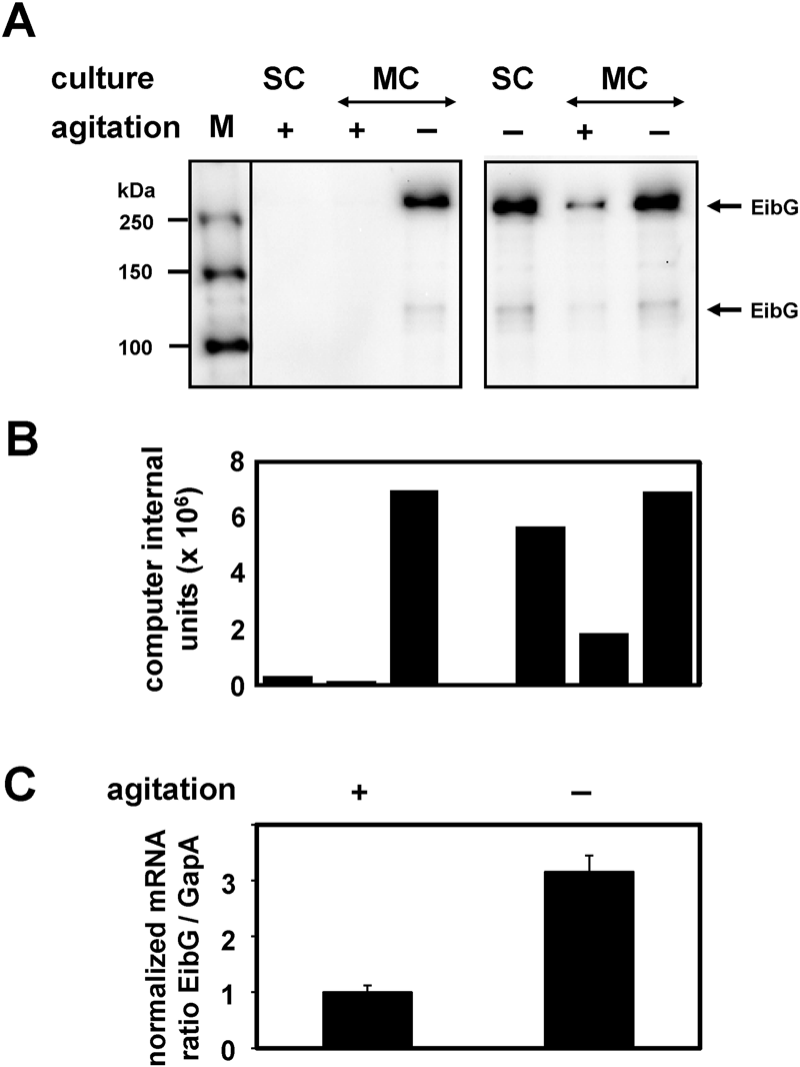
EibG expression is regulated by motion. Single colonies of strain 2875/96 were grown as starting culture (SC) in LB for 18 h with (+) or without (-) shaking. Cells were inoculated at a 1:100 dilution indicated as main culture (MC) to fresh LB medium followed by incubation with or without shaking, respectively. Proteins of grown cells (1 μg per lane) were separated by SDS-PAGE and immunoblotted. (A) EibG signals were visualized using human IgG Fc fragment conjugated to HRP. (B) EibG signals were quantified densitometrically and are reported as computer internal units. Values presented are representative of those from repeated growth conditions and independent gel runs. (C) After inoculation RNA was isolated for RT-PCR. The bars represent the relative levels of EibG mRNA normalized to GapA expression. Values for bacteria grown under agitated conditions (+) were defined as 1.0 and the increase of EibG mRNA was calculated after static growth (-). Samples were analysed in triplicate and results represent the means and standard deviations of the mean.

The varying EibG protein regulation was confirmed under agitated and static growth conditions; hence strains were pre-cultured with and without shaking. To determine if this regulation is reversible, i.e., does a highly expressing strain produce scant or no EibG when shaken, we re-inoculated agitated and non-agitated starter cultures into fresh medium and incubated again with and without shaking (Fig. 3A, B). Strain 2875/96 demonstrated high EibG levels under motionless growth conditions and moderate or signals at the detection limit with agitation. The same phenotypes were recapitulated. EibG signals hyper-expressed after static growth decreased dramatically under agitation but remained at a high level when grown statically. On the other hand, poorly expressed EibG signals increased when strains were incubated under static conditions. Taken together, bacteria grown under agitated conditions produced low levels of EibG, and bacteria grown without agitation produced abundant EibG, recapitulating the initial finding of the impact of agitation on EibG expression.

This up-regulation with increased EibG expression was also demonstrated on the RNA level with an increase in the relative abundance of *eib*G mRNA. The *eib*G mRNA signal rose by a factor of 3.16 compared with the mRNA level of the constitutively expressed *gap*A (Fig. 3C). This result suggests a regulatory effect on EibG expression by motion and static growth.

### Role of EibG

**Fig 4:**
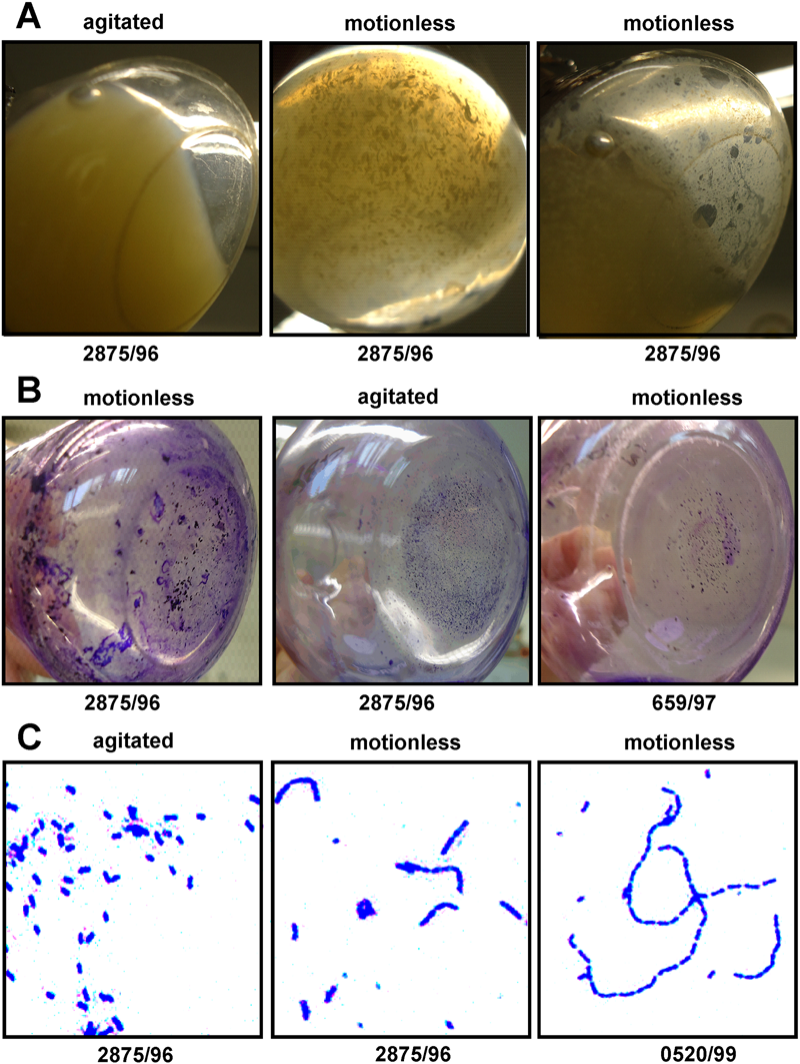
Phenotypes of EibG-strains after agitation and static growth. Strain 2875/96 was inoculated for 20h at 37°C with and without shaking. Bacteria grown under agitated conditions grew homogenously and were turbid without biofilm formation (A). However, statically grown bacteria aggregated and deposited a biofilm (A). Aggregates and deposits were stained with crystal violet (B). Strain 659/97 was used as EibG negative control. Microscopically, shaken bacteria demonstrated single and non-aggregated cells whereas static grown bacteria formed coherent chains (C). The figures exemplify microscopic images of strain 2875/96 and 0520/99, magnified as indicated.

Bacteria that express EibG demonstrate a chain-like adherence pattern to host epithelial cells [13, 30]. Agitated overnight bacterial cultures demonstrated homogenous growth with consistent turbidity (Fig. 4A), while statically grown bacteria formed clumps. A biofilm of the EibG, shown here for strain 2875/96, was detected as a deposit on the inoculation tube under static but not agitated growth (Fig. 4B) for EibG expressing wild-type strains. While cell chains formed under static growth, most bacterial cells remained solitary after agitated growth (Fig. 4C). Bacteria carrying cloned EibG-types produced biofilms as well; however the cell aggregation was less pronounced (data not shown). If any, very few chain formations were detectable. However, after static growth, increased numbers of chains were identified. These differential phenotypes were induced by static growth versus agitation, related to organisms with EibG of the α-type without (strain 2875/96) and with (strain 3671/97) EibC, and EibG of the γ-type (strain 0520/99) (Table 3). These data indicate a direct association between phenotype and EibG expression, with enhanced chain formation of EibG strains with growth conditions that augment EibG expression.

**Table 3:** Phenotypical characteristics of EibG expression.

phenotype	strain no.	agitation	motionless
Biofilm formation	3671/97	-	+
	2875/96	-	+
	0520/99	-	+
	659/97	-	-
Chain formation	3671/97	-	+
	2875/96	-	+
	0520/99	-	+
	659/97	-	-
Aggregation	3671/97	-	+
	2875/96	-	+
	0520/99	-	+
	659/97	-	-

**Fig 5:**
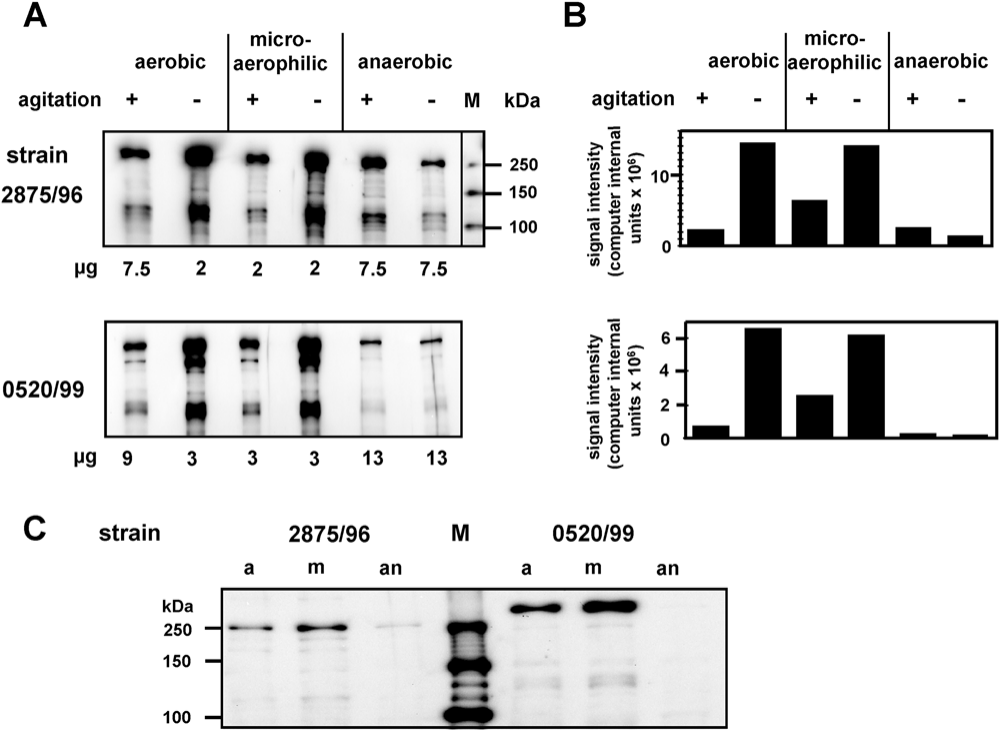
Reduced oxygen up-regulates EibG expression. Strains 2875/96 and 0520/99 were inoculated into agitated (+) or static conditions (-) in jars under aerobic, microaerophilic and anaerobic conditions at 37°C for 18h. After cell harvesting, proteins were loaded and immunoblotted. (A) EibG proteins were visualized by chemiluminescence and (B) EibG signals were densitometrically quantified and are reported as computer internal units. Values are typical of those from repeated growth conditions and independent gel runs. (C) Cells were harvested from LB plates. Proteins of 5 and 15 μg per lane were loaded for 2875/96 and 0520/99, respectively, and EibG proteins were immunologically detected.

As shown, EibG proteins are highly regulated in expression with high levels after static growth and very low levels after growth conditions with agitation. To determine if shear stress plays the only major role for the up- and down-regulation we analyzed the influence of oxygen in the next experimental set-up, because bacteria grown under agitated conditions are exposed to higher oxygen concentration than bacteria grown under static conditions. Cultures of 2875/96 (α-type) and 0520/99 (γ-type) were inoculated with and without agitation under aerobic, microaerophilic and anaerobic conditions (Fig. 5). Bacteria produced high EibG signals under aerobic and microaerophilic growth without agitation while faint but visible bands were detected with shaking (Fig. 5A). Interestingly, aeration may have an appreciable influence on the EibG expression level under agitation, as protein signal intensities were considerably higher under microaerophilic than under aerobic growth proven by signal quantification (Fig. 5B). In contrast, EibG expression was at the detection limit in each case when cultures were inoculated under anaerobic growth independent on static or shaking conditions. To verify this regulatory effect we cultivated the strains on plates followed by aerobic, microaerophilic and anaerobic inoculation (Fig. 5C). Increased EibG expression was observed under microaerophilic growth conditions, less intense signals were detected with aerobic growth, and only very faint bands were visible with anaerobic growth.

**Fig 6:**
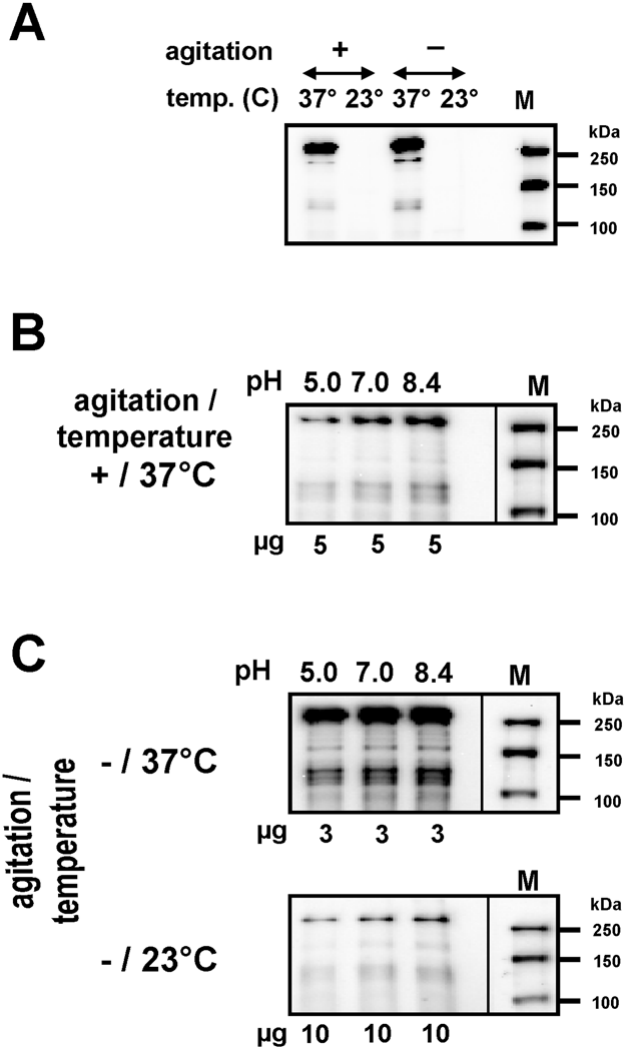
Temperature and pH have an influence on EibG expression. Bacteria were incubated in LB with different pH values as indicated at 37°C and 23°C with agitation (+) and under static growth conditions (-). Proteins were immunoblotted and EibG signals were measured using chemiluminescence. The influence of temperature (A), pH values (B) and both in combination (C) are shown for wild-type strain 2875/96. Marker sizes (M) are provided.

Low temperature provoked a decrease of EibG synthesis to the limit of detection, independent of static or agitation conditions (Fig. 6A). Growth in alkaline LB media elicited a modest increase in EibG expression whereas neutral and acid pH decreased EibG levels (Fig. 6B). Taken together, highest EibG levels were detected in static grown cultures at 37°C and alkaline pH while EibG proteins were repressed in acid cultures grown under shaking conditions at 23°C (Fig. 6C).

## Discussion

We demonstrated that EibG protein expression in *E*. *coli* is mainly influenced by static and agitated growth conditions. When grown under shaking conditions, bacteria down-regulate EibG, and cultures synthetized more EibG when grown under static conditions, and this phenotype correlates with bacterial aggregation. The up-regulation of EibG under static growth conditions contrasts with expression of other bacterial adhesins [31–33]. Additional regulatory effects for increased EibG expression were found with oxygen reduction, alkaline pH and a high temperature at 37°C.

EibG proteins occur mostly as multimers with molecular masses > 250 kDa under the conditions employed in this study. Multimerized proteins of the Eib family are suggested as an additional putative afimbrial adhesin in STEC that might be involved in mammalian cell host-pathogen interactions [13, 30, 34].

EibG proteins appear in three isoforms (α-, β- and γ-subtypes) [13]. Although the lengths of the gene products are similar, γ-subtypes vary at up to 61 amino acid changes compared to the α-subtype, but the protein bands of the γ-type have higher molecular masses in immunoblots, suggesting varying degrees of multimerization. The subtypes of the EibG proteins seem to be involved directly in the generation of differential appearance of chains by growing bacteria. However, EibG over-expression in static cultures of wild-type strains produced elongated chains, whereas chain formation was scant by bacteria grown in shaken cultures.

The subcloning of *eib* genes into multicopy plasmids to measure protein expression has been employed for genetic and molecular investigations [11, 13]. However, recombinant proteins do not provide naturally expressed and regulated Eib proteins, and the pertinence of data from over-expressed proteins is not established. Specifically, post-translational modification of proteins (e.g., folding) might vary from what occurs with naturally expressed proteins. Also, overexpression might produce non-physiologic aggregation manifest as inclusion bodies [35]. Finally, the study of the in-host (animal and human) regulation of protein expression and therefore of natural biological function of Eib is precluded. Complex biological analysis is optimally performed with naturally expressed proteins, even if they are expressed under restricted conditions, in this case, non-agitated growth.

Eib proteins might represent a type V autotransporter (AT) secretion system. Common features of ATs are an amino terminal signal sequence, a passenger, and a translocation domain [36]. ATs belong to three groups: the serine protease AT in *E*. *coli* (SPATEs), the AIDA-I type, and the trimeric autotransporter adhesins (TAAs), which include the Eib-ATs. Multiple *eib* genes in *E*. *coli* strains are associated with immunoglobulin binding and serum resistance [10, 11]. Specific subtypes interact with each other generating mixed multimers or a hetero-multimerization with other Eib subtypes or with other proteins [37]. Such natural expression might lead to different binding affinities. Several TAAs demonstrate similar phenotypes in *E*. *coli* as the Saa protein [38], an STEC agglutinating adhesin which is coded on a mega plasmid inducing adhesion to Hep-2 cells and autoaggregation. Furthermore, adherence to T24 cells, autoaggregation and biofilm formation is associated with UpaG [39]. We have demonstrated that the EibG protein confers an adhesive (to eukaryotic cells) phenotype [13], and forms bacterial chains [30] and wild-type strains yield in aggregation. However, EibG expressing wild-type strains additionally grow surface attached in biofilms. This phenotype however was not observed in this way when EibG was expressed from sub-cloned *eibG*, indicating that other factors which are regulated and expressed in wild-type strains might have an additional impact on this phenotypic expression.

Further studies of the expression of this protein might provide insights into biological functions of the proteins and their interaction with similar or conversely reacting proteins. It is difficult to know the exact circumstances bacteria encounter in human or animal hosts, but highly agitated growth of EibG-producing pathogens, in pure culture, is probably an unrealistic condition. In any event, the interplay between gene regulation, growth conditions, physic-chemical and mechanical conditions, and corresponding pathogen protein expression in the colonized host is an area worthy of further pursuit.
